# Bispecific killer cell engager with high affinity and specificity toward CD16a on NK cells for cancer immunotherapy

**DOI:** 10.3389/fimmu.2022.1039969

**Published:** 2023-01-06

**Authors:** Shahryar Khoshtinat Nikkhoi, Geng Li, Suha Eleya, Ge Yang, Venu Gopal Vandavasi, Arash Hatefi

**Affiliations:** ^1^ Department of Pharmaceutics, Rutgers University, Piscataway, NJ, United States; ^2^ Department of Chemistry, Biophysics Core Facility, Princeton University, Princeton, NJ, United States; ^3^ Cancer Pharmacology Program, Cancer Institute of New Jersey, Rutgers University, New Brunswick, NJ, United States

**Keywords:** bispecific killer cell engager, BiKE, NK92 cells, CD16a, VHH nanobody, cancer immunotherapy, HER2, ovarian cancer

## Abstract

**Introduction:**

The Fc region of monoclonal antibodies (mAbs) interacts with the CD16a receptor on natural killer (NK) cells with “low affinity” and “low selectivity”. This low affinity/selectivity interaction results in not only suboptimal anticancer activity but also induction of adverse effects. CD16a on NK cells binds to the antibody-coated cells, leading to antibody-dependent cell-mediated cytotoxicity (ADCC). Recent clinical data have shown that the increased binding affinity between mAb Fc region and CD16a receptor is responsible for significantly improved therapeutic outcomes. Therefore, the *objective* of this study was to develop a bispecific killer cell engager (BiKE) with high affinity and specificity/selectivity toward CD16a receptor for NK cell-based cancer immunotherapy.

**Methods:**

To engineer BiKE, a llama was immunized, then high binding anti-CD16a and anti-HER2 VHH clones were isolated using phage display. ELISA, flow cytometry, and biolayer interferometry (BLI) data showed that the isolated anti-CD16a VHH has high affinity (sub-nanomolar) toward CD16a antigen without cross-reactivity with CD16b-NA1 on neutrophils or CD32b on B cells. Similarly, the data showed that the isolated anti-HER2 VHH has high affinity/specificity toward HER2 antigen. Using a semi-flexible linker, anti-HER2 VHH was recombinantly fused with anti-CD16a VHH to create BiKE:HER2/CD16a. Then, the ability of BiKE:HER2/CD16a to activate NK cells to release cytokines and kill HER2^+^ cancer cells was measured. As effector cells, both high-affinity haNK92 (CD16^+^, V176) and low-affinity laNK92 (CD16^+^, F176) cells were used.

**Results and discussion:**

The data showed that the engineered BiKE:HER2/CD16a activates haNK92 and laNK92 cells to release cytokines much greater than best-in-class mAbs in the clinic. The cytotoxicity data also showed that the developed BiKE induces higher ADCC to both ovarian and breast cancer cells in comparison to Trazimera™ (trastuzumab). According to the BLI data, BiKE:HER2/CD16 recognizes a different epitope on CD16a antigen than IgG-based mAbs; thus, it provides the opportunity for not only monotherapy but also combination therapy with other antibody drugs such as checkpoint inhibitors and antibody-drug conjugates. Taken together, the data demonstrate the creation of a novel BiKE with high affinity and specificity toward CD16a on NK cells with the potential to elicit a superior therapeutic response in patients with HER2^+^ cancer than existing anti-HER2 mAbs.

## Introduction

The crystallizable fragment (Fc) of IgG-based monoclonal antibodies binds to Fcγ receptors (FcγRs) that are expressed on the surface of leucocytes and required for the efficacy of most antibody drugs. The FcγRs are divided into two main categories of activating and inhibitory, which bind to Fc regions of antibodies with different affinities. Activating receptors include FcγRI/CD64, FcγRIIa/CD32a, FcγRIIc/CD32c, FcγRIIIa/CD16a, and FcγRIIIb/CD16b and the inhibitory ones include FcγRIIb/CD32b (1). CD16a, a low-affinity receptor, is the primary FcγR on the surface of natural killer (NK) cells that binds to monoclonal antibodies (mAbs). CD16a on NK cells binds to the antibody-coated cells (e.g., cancer cells), triggering antibody-dependent cell-mediated cytotoxicity (ADCC). Owing to this function, CD16a-expressing NK cells are currently being investigated in clinical trials for cancer therapy (e.g., NCT04673617 and NCT03383978). Recent clinical data have shown that the increased binding affinity between mAb Fc region and CD16a receptor is responsible for the significantly improved therapeutic outcomes (2–4). In addition to affinity, the selective binding of antibodies with CD16a also plays a significant role in boosting the therapeutic efficacy and reducing the off-target toxicities. For example, due to a phenomenon, called sink condition, antibodies that bind to FcγRIIIb/CD16b receptor on neutrophils have been shown to restrict the ADCC activity of NK cells against cancer cells (5). Furthermore, unwanted binding of mAbs to inhibitory FcγRIIb/CD32b (expressed on B cells and macrophages) has been shown to inhibit B cell maturation and macrophage activation (6, 7). CD32b is also expressed on a subset of CD8^+^ T cells, where its activation reduces T cell survival by activating Caspase 3 and 7 pathways leading to decreased T cell-based immunity (8). Consequently, several groups are striving to engineer antibodies with a high affinity toward CD16a receptor without cross-reactivity with CD16b and/or CD32b (9, 10). Therefore, the *objective* of this study was to engineer a functional bispecific killer cell engager (BiKE) with high affinity and specificity/selectivity toward CD16a receptor. To achieve our objective, we took advantage of a unique class of single-domain antibodies, known as VHH or nanobody, which was first found in the blood of camelidae family (11, 12). We immunized llama with recombinant human CD16a protein (rCD16a) and rHER2 to make it generate anti-CD16a and anti-HER2 VHHs. Then, using phage display, we isolated an anti-CD16a VHH clone with high affinity toward CD16a without cross-reactivity with CD16b-NA1 or CD32b. Similarly, anti-HER2 VHH clone with high affinity and specificity/selectivity toward HER2 without cross-reactivity with HER1, HER3, and HER4 was isolated. The affinities of the VHHs were measured using biolayer interferometer (BLI), and the cross-reactivity was determined by ELISA and flow cytometry. Using a semi-flexible linker, HER2 VHH was recombinantly fused with CD16a VHH to engineer BiKE:HER2/CD16a. Subsequently, the ADCC of BiKE:HER2/CD16a against HER2^+^ cancer cells was measured using both haNK92 (CD16^+^, V176) and laNK92 (CD16^+^, F176) cells as effector cells and compared with Trazimera™ (trastuzumab). Trastuzumab is an FDA-approved mAb against HER2 where its Fc region interacts with CD16a receptor with low affinity and selectivity. Here, we used NK92 cell line because it is available as both high-affinity (CD16^+^, V176) and low-affinity (CD16^+^, F176) variants, is FDA-approved for human use, and has a broad range of anticancer activity. The ability of BiKE:HER2/CD16a to activate NK92 cells was determined by inspecting the CD107a degranulation and measuring the release of cytotoxic proteins and cytokines (i.e., Perforin, Granzyme B, TNF-α, and IFN-γ) using flow cytometry and ELISA, respectively.

## Materials and methods

### Materials used for the generation, isolation, and characterization of VHHs and BiKE

The list of cells, antibodies and reagents used in this study are provided in **Supplementary Table S1**.

### Cell lines and culture

CD16a^+^ haNK92 (V176) and CD16a^+^ laNK92 (F176) cells were purchased from ATCC and cultured in α-MEM supplemented with 12.5% Fetal Bovine Serum (FBS), 12.5% Horse Serum, 0.2 mM myo-inositol, 0.02 mM Folic Acid, 100 U/ml penicillin-streptomycin, 0.1 mM 2-Mercaptoethanol, and 100 U/ml IL-2. BT474 (HER2^+^ breast cancer) was purchased from ATCC and maintained in Hybri-Care Media supplemented with 10% FBS and 100 U/ml penicillin-streptomycin. JIMT-1, HER2^+^ breast cancer cell line resistant to anti-HER2 antibodies (13), was kindly provided by Dr. Jason S. Lewis (MSKCC, NY) and cultured in DMEM/F-12, GlutaMAX™ supplemented with 10% FBS, 100 U/ml penicillin-streptomycin, and 10 µg/ml insulin. SKOV-3 (HER2^+^ ovarian cancer) cell line was obtained from ATCC and maintained in McCoy’s 5A (Modified) Medium supplemented with 10% FBS. Neutrophils were purchased from HemaCare and cultured in DMEM-10% FBS for an hour, then immediately used for downstream application. B cells were purchased from Charles River and cultured in RPMI-1640 supplemented with 10% fetal calf serum (FCS) and 2 mM L-Glutamine.

### Expression and purification of rCD16a and rHER2 proteins

The genes encoding rCD16a (UniProt ID P08637) and rHER2 ectodomain (Uniprot ID P04626) were synthesized by VectorBuilder (IL, USA) and then cloned into two separate PiggyBac mammalian expression vectors under EF-1α promoter. The mammalian expression system was selected because the rCD16a ectodomain is a glycosylated protein. A secretory signal sequence was designed at the proteins’ N-terminal and a His-Tag at their C-terminal to facilitate purification from the culture media by Ni-NTA column chromatography (**Supplementary Tables S2, S3**). To express rCD16a and rHER2, FreeStyle™ 293-F cells were cultured per manufacture’s protocol using a 250 ml Reusable Spinner flask. Cells were passaged every three days. On the day of transfection, cells were seeded at ~3 × 10^6^ cells/ml in 100 ml of FreeStyle™ 293 expression media using 250 ml Reusable Spinner Flask for 30 min. The PiggyBack expression vectors were then complexed with linear polyethylenimine (PEI) 25K. For every 10^6^ cells, 1 µg plasmid was mixed with 4 µg of PEI (1:4 w/w ratio) in Opti-MEM and incubated at room temperature (RT) for 20 minutes. Next, the plasmid:PEI complexes were added to the seeded cells dropwise under constant stirring. The flask was incubated at 37°C with 5% CO_2_ for 24h. The next day, two-fold fresh FreeStyle media was added to the transfected cells. Protein expression continued for 8 to 10 days, or until the viability dropped below 85%, whichever came first. To extract and purify proteins, cells were centrifuged at 10,000 g, 10 min, 4°C, followed by supernatant collection and filtration through a 0.45 µm filter to remove the cell debris. Then, 300 µL of Ni-NTA resin was equilibrated with buffer (500mM NaCl, 20mM Na_2_HPO_4_, 50mM Tris, pH 7.4), mixed with the supernatant, and incubated at 4°C overnight. The next day, the supernatant/Ni-NTA mixture was poured onto a chromatography column and washed using 20 ml of washing buffer (500mM NaCl, 20mM Na_2_HPO_4_, 50mM Tris, 7.5 mM imidazole, pH 7.4). Finally, rCD16a and rHER2 proteins were eluted using 500 µl of elution buffer (500mM NaCl, 20mM Na_2_HPO_4_, 50mM Tris, 250mM imidazole, pH 7.4). The purity and molecular weight of the purified proteins were estimated by sodium dodecyl sulfate–polyacrylamide gel electrophoresis (SDS-PAGE).

### Immunization of llama with rCD16a and rHER2 proteins

Llama immunization was carried out by Capralogics Inc. (Gilbertville, MA). A female llama was immunized *via* injections every two weeks for a total of six injections using rCD16a or rHER2 (500 µg/injection) (**Supplementary Figure S1**). The antigen of interest was mixed with either complete (first injection) or incomplete Freund’s adjuvant (second, third and fifth injections) to maximize the immune response. The fourth and sixth injections were performed using the proteins without any adjuvant. After the fourth injection, 50 ml of whole blood was collected, and the immunization was confirmed by ELISA using commercial CD16a (F176) and HER2 proteins. Then, llama immunization was continued for four more weeks. Five days after the sixth injection, 600 ml of whole blood was withdrawn to isolate the peripheral blood mononuclear cells (PBMCs), which were purified by Ficoll-Hypaque using density gradient centrifugation method (400g, 30 min, RT) (14).

### Library generation

After collecting and counting the PBMCs using the Trypan Blue Exclusion method, total RNA was extracted using RNeasy Mini Kit followed by cDNA synthesis using oligo-dT and random hexamer *via* SuperScript™ IV First-Strand Synthesis system. After that, the generated cDNA was used as a template in the Nested PCR reaction using a set of primers (**Supplementary Table S4**) and PCR protocol (**Supplementary Table S5**). The band representing VHHs gene (400 bp) was cloned into pMECS-GG phagemid (Kindly provided by Dr. S. Muyldermans, Belgium) using the Golden Gate cloning system. Finally, the recombinant phagemid was used to transform TG1 E. coli (Electroporator setting: 2.5 kV, 25 µF, 200 Ω). The transformants were selected in six flasks of 100 ml Luria-Bertani (LB) supplemented with 100 µg/ml carbenicillin. The propagated bacteria were pooled, spun down, and resuspended in 20 ml of fresh LB media. After adding 15% glycerol, the library was stored at -80°C for further use.

### Phage display

Four rounds of phage display were performed to reach specific anti-CD16a and anti-HER2 libraries. First, 2 OD_600nm_ (1 OD_600nm_ = 2.66 × 10^9^ cells/ml) of llama library was inoculated into 100 ml of 2xYT supplemented with 100 µg/ml carbenicillin and 2% Glucose. Once the OD_600nm_ reached 0.6, 500 µl of the helper phage, VCSM13, was added to the culture and incubated at 37˚C without shaking for 60 min. The infected bacteria were harvested by centrifugation (10,000 g, 20 min, 4˚C) and supernatant was discarded. The pellet was resuspended in 100 ml of 2xYT (without Glucose), supplemented with 100 µg/ml carbenicillin and 50 µg/ml kanamycin, and incubated at 37˚C overnight with shaking. The next day, bacteria were spun down at speed of 10,000 g for 20 min at 4˚C. Then, 10 ml of PEG6000 (20% W/V)/2.5 M NaCl solution was added to the 40 ml supernatant and kept on ice for 60-120 minutes. Finally, the recombinant phages were recovered *via* centrifugation (3,000 g, 20 min, at 4˚C) followed by washing three times with DPBS.

The purified phages were used in polyclonal phage ELISA. In this experiment, all the incubations were at room temperature (RT) and a shaking speed of 700 rpm unless mentioned otherwise. The bait protein (either CD16a or HER2) was coated into a 96-well plate overnight at 4˚C. The next day, after blocking with 2% skimmed milk, ~100 µl of recombinant phages were added and incubated at RT for 30 min. To remove the non-specific VHHs, the plate was washed ten times with 0.1% DPBS-T followed by ten times washing with DPBS. Finally, the binders were recovered using 50 µl of 0.25% Trypsin (enzymatic elution), which was neutralized by 4-(2-Aminoethyl) benzene-1-sulfonyl fluoride (AEBSF). The purified phages were used in equal quantities to infect 100 µl of TG1 bacteria for one hour at 37˚C, which was then transferred into 100 ml of LB media, supplemented with 100 µg/ml carbenicillin, followed by overnight bacterial culture.

### Preparation of periplasmic extract and evaluation of affinity and selectivity by ELISA

To select the top binders, 190 colonies were randomly selected to be cultured in 1 ml of LB media, supplemented with 100 µg/ml carbenicillin and 2% Glucose, in a Deep-Well 96-well plate. To keep the culture oxygenated, a ventilated adhesive plate was used to cover the plate. The following day, 1 ml of fresh LB media, supplemented with 100 µg/ml carbenicillin, was inoculated with 10 µl of overnight culture. After four hours, the expression was induced using 1 mM Isopropyl β- d-1-thiogalactopyranoside (IPTG). The expression continued overnight at 28°C. The next day, the bacteria were harvested using centrifugation (10,000 g, 7 min, 4˚C). The supernatant was discarded and the pellet was stored at -20°C. Next, the periplasmic extract was prepared using three cycles of freeze-thaw (30 min at -20°C, 10 min at RT). Next, 500 µl of DPBS was added to the pellet and placed on a shaker for 30 min following centrifugation (10,000 g, 30 min, 4°C). Finally, the supernatant (400 µl) was transferred to a new Deep-Well 96-well plate. The periplasmic extract was used in ELISA to find the top binding candidates.

For anti-CD16a ELISA, the plate was coated using 100 µl of 5 µg/mL streptavidin overnight at 4°C. The next day, after one round of washing, 50 µl of biotinylated CD16a and CD16b were added and incubated for 30 minutes. For anti-HER2 ELISA, 100 µl of 1µg/ml HER2 protein and three other members of the Heregulin superfamily, including HER1, HER3, and HER4 were used to coat Nunc MaxiSorp™ high protein-binding capacity 96-well ELISA plate and incubated overnight at 4°C. After three times washing, 100 µl of the periplasmic extract was added. To detect the bound VHH, the secondary antibody, HRP anti-HAtag antibody (1:10000 dilution) was added. To develop color, 50 µl of 1-Step™ Turbo TMB-ELISA substrate solution was added and incubated in darkness for 15 min. After stopping the reaction using 50 μL of 1N HCl, the plate was read at 450 nm wavelength using Tecan Infinite^®^ M200 PRO plate reader.

### Expression and purification of VHHs and BiKE

Anti-HER2 and anti-CD16a VHHs were sequenced by GeneWiz (NJ, USA) and then cloned into pHEN6 (Kindly provided by Dr. S. Muyldermans, Belgium) expression vectors. The pHEN6 was chemically transformed into WK6 E. coli expression system, followed by the selection of the highest expressing transformant using western blot analysis. Next, 750 ml of Terrific Broth (TB), supplemented with 0.1% Glucose, 1 mM MgCl_2_ and 100 μg/ml carbenicillin, was inoculated by 7.5 ml of overnight culture grown at 37°C. After the OD_600nm_ reached 0.6-0.8, 0.5 mM IPTG was added to induce expression. The culture was then incubated at 28˚C overnight. The next day, the media was spun down using a centrifuge (10,000 g, 10 min, 4˚C), the pellets were resuspended in 10 ml of TES buffer (200 mM Tris–HCl, pH 8.0, 500 mM sucrose, 1 mM EDTA), and incubated at 4˚C for one hour while shaking. Next, 15 ml of TES/4 buffer (10 ml TES buffer was added to 30 ml distilled water) was added and incubated at 4˚C for 45 min while shaking. The bacteria were pelleted *via* centrifugation (40,000 g, 4°C, 30 min) and the supernatant was loaded onto the Ni-NTA column. The column was washed with 30 ml of wash buffer (500 mM NaCl, 20 mM Na_2_HPO_4_, 50 mM Tris, 25 mM imidazole, pH 7.4). The purified VHH was eluted using elution buffer (500 mM NaCl, 20 mM Na_2_HPO_4_, 50 mM Tris, 500 mM imidazole, pH 7.4). The purity of the eluted VHH was evaluated by SDS-PAGE.

To express BiKE:HER2/CD16a, the gene encoding the construct was designed as a single chain peptide by fusing anti-CD16a VHH (C1 clone) with anti-HER2 VHH (E5 clone) *via* HMA (human muscle aldolase) as a linker. WELQut protease site, c-myc, and His-Tag sequences were designed at the BiKE’s C-terminal to facilitate its purification and detection. The BiKE:HER2/CD16a (E5C1) construct was then synthesized and cloned into a pHEN6 expression vector by GenScript (Piscataway, NJ), followed by transformation into WK6^®^ *E. coli* expression host. Next, 750 ml of Terrific Broth (TB) containing 100 μg/ml carbenicillin was inoculated with 7.5 ml of overnight culture. After 16 – 18h, when the OD_600nm_ reached ~5, 1 mM IPTG was added to induce protein expression. The expression continued at 37°C for three hours and the bacterial culture was centrifuged at 10,000 g for 10 min at 4°C). The pellet was resuspended in lysis buffer (1 M NaCl, 100 mM KCl, 50 mM Tris, 20 mM Phosphate Buffer, 0.01% Tween 20, 15 mM imidazole, pH 8) (3 ml per gram of pellet) and incubated at 4°C for 30 min while shaking. The bacterial suspension was then sonicated (5s on, 3s off, 70% amplitude) for 30 min on ice. Next, the cell debris was pelleted *via* centrifugation (40,000 g, 4°C, 30 min), the supernatant was loaded onto a Ni-NTA column and washed with 30 ml of wash buffer (500 mM NaCl, 100 mM KCl, 100 mM Tris, 20 mM Phosphate Buffer, 25 mM imidazole, pH 8). To elute BiKE, the column was equilibrated with 20 ml of DPBS and then eluted using 10 U/mL of WELQut protease. The purity of the eluted protein was measured using SDS-PAGE. The molecular weight of the purified BiKE and its monomeric status was evaluated at Rutgers Center for Advanced Biotechnology and Medicine core facility using liquid chromatography/mass spectroscopy (LC-MS).

### Evaluation of the selectivity of anti-CD16a VHH, anti-HER2 VHH, and BiKE by ELISA

The purified VHHs were subjected to functional analysis using ELISA. For anti-CD16a VHH ELISA, 50 µl of biotinylated CD16a, CD16b-NA1, CD16b-NA2, and CD32b proteins were added to the streptavidin-coated plate and incubated for 45 min at RT. Similarly, for anti-HER2 VHH ELISA, HER2, HER1, HER3, and HER4 proteins were used to coat ELISA plates. Following washing, 100 µl of 100 nM purified VHHs were added and incubated at RT for one hour. Next, 100 µl anti-HA tag HRP conjugated secondary antibody was added. Finally, the substrate was added for color development, and the plate was read at OD450nm.

The binding of purified BiKE:HER2/CD16a to HER2 and CD16a antigens was also evaluated using ELISA. For this purpose, 50 µl of biotinylated CD16a or HER2 was added to the streptavidin-coated plate and incubated for 45 min at RT. Followed by washing and blocking, 100 µl of purified VHH or BiKE ranging from 1000 to 0 nM were added and incubated at RT for one hour. Next, 100 µl anti-cMyc tag HRP conjugated secondary antibody (1:10,000 dilutions) was added and the plate was read at OD450 nm.

### Evaluation of the ability of anti-HER2 VHH and BiKE to recognize HER2^+^ cells

HER2^+^ SKOV-3 and HER2¯ MDA-MB-231 cancer cells were cultured followed by incubation with the purified anti-HER2 VHH or BiKE. Approximately 10^6^ cells were counted and washed using DPBS-1% FBS. Then, equivalent to 100 nM of trastuzumab, pertuzumab, anti-HER2 VHH, or BiKE was added to the cells and incubated on ice for 60 min. After the washing step, FITC-conjugated anti-Human IgG or anti-Histag antibody was added and incubated on ice for 60 min. Cells were washed three times and then analyzed using Beckman Coulter Gallios flow cytometer at Rutgers Flow cytometry core facility.

### Evaluation of the ability of anti-CD16a VHH and BiKE to recognize CD16a^+^ cells

NK92 cells (CD16a^+^), neutrophils (CD16b^+^), and B cells (CD32b^+^) were cultured according to the vendors’ protocols. Cells were harvested and washed once with DPBS supplemented with 2% FBS (2% DPBS-FBS). Next, the non-specific interaction of antibody Fc region with Fc receptors was blocked using Human TruStain FcX™ to eliminate background staining.

To evaluate the selectivity of anti-CD16a VHH (clone C1) toward CD16a antigen, 0.5 x 10^6^ NK92 cells (CD16a^+^) and neutrophils (CD16b^+^) were stained with 3G8 mAb and C1 VHH on ice for 60 min. Cells were washed three times and incubated on ice with PE-conjugated Rabbit anti-mouse antibody and anti-HisTag antibody for 45 min to label bound 3G8 and C1 VHH, respectively. Samples were then analyzed by Beckman Coulter CytoFLEX Flow Cytometer. The same method was used to evaluate the ability of BiKE to recognize CD16a on NK92 cells.

To evaluate the interaction of anti-CD16a VHH (clone C1) with CD32b on B cells, B Cells were stained with AT10 (Anti-CD32a/b mAb) and C1 VHH, as mentioned above. Next, cells were stained with FITC-conjugated Rabbit anti-mouse antibody and anti-HisTag antibody to label bound AT10 and C1 VHH, respectively. Samples were then analyzed by Beckman Coulter CytoFLEX Flow Cytometer.

### Measurement of VHH and BiKE binding affinities and kinetics using Biolayer Interferometer

To evaluate the binding affinity (K_D_) and constant rates of association (Kon) and dissociation (Koff) of VHHs and BiKE, an Octet RED96e (Sartorius) Biolayer Interferometer (BLI) located at Biophysics Core Facility at Princeton University was used. An Octet^®^ Streptavidin (SA) Biosensor was soaked for at least 10 min in DPBS supplemented with 0.1% Casein, biotinylated CD16a, CD16b-NA1, CD16b-NA2, and HER2 antigens were loaded onto the streptavidin (SA) biosensor until 1 nm shift was reached. Next, the sensor was dipped into the washing buffer (DPBS + 0.05% Tween 20 + 0.1% Casein) for 2 min to reach the baseline. Then, the sensor was submerged into wells containing 60, 30, 15, 7.5, 3.75, 1.875, and 0 nM of purified anti-CD16a VHHs or BiKE for 5 min (association step). Then, in the dissociation step, sensors were dipped into the washing buffer for 5 min to acquire data (**Supplementary Table S6**). The data were then analyzed using Octet Data Analysis HT 11.1 software (Biophysics Core Facility at Princeton University). The sensorgrams were subtracted from the reference and fitted into 1:1 binding model for data analysis. Finally, the affinity and kinetics were analyzed using “Association and Dissociation”.

### Evaluation of the ADCC by a cell toxicity assay

The ability of BiKE to induce ADCC was evaluated and compared with mAb trastuzumab. In this experiment, target cancer cells (i.e., SKOV-3, BT474, and JIMT-1) were under either adherent or non-adherent conditions. To do ADCC under the adherent condition, 10^4^ cancer cells were seeded in a tissue culture treated 96-well plate. The next day, 100 nM of BiKE or mAb was added to cells and incubated at 37°C for 30 min. Next, laNK92 or haNK92 cells were added to target cells to make E:T ratios of 4, 2, 1, 0.5, 0.25, and 0 and incubated for four hour at 37°C. Next, the wells were washed twice using DPBS to remove NK92 cells. Finally, 10% alamarBlue ™ HS Cell Viability Reagent was added to the plate and incubated for one to two hours at 37°C. Then, the plate was read using Tecan Infinite M Plex plate reader and the cell viability was calculated.

To do ADCC under the non-adherent condition (in suspension), 5×10^3^ target cancer cells were mixed with 100 nM of mAb or BiKE and then added to a non-treated 96-well plate. Then, different numbers of haNK92 cells were added to each well to obtain the E:T ratios of 16, 8, 4, 2, 1, 0.5, and 0. The plate was incubated at 37°C for four hours. Next, alamarBlue ™ HS Cell Viability Reagent was added and incubated at 37°C for two to three hours. Finally, the plate was read by a Tecan Infinite M Plex plate reader and the cell viability was measured using the following formula:

[(Fluorescent Intensity of Test Group - Fluorescent Intensity of NK only)/(Fluorescent Intensity of Target Cells only)] × 100

### Evaluation of the release of cytokines and cytotoxic proteins from NK92 cells during ADCC

SKOV-3 cells were seeded in a tissue culture treated 96-well plate at the density of 10,000 cells per well. The next day, 10 nM BiKE or trastuzumab was added to the plate and incubated at 37˚C for 30 min. Next, effector cells (laNK92 or haNK92) were added at E:T ratio of 4. After two hours (Perforin and Granzyme B) and 24h (TNF-α and IFN-γ), plates were centrifuged down at 2000 g for 10 min to pellet the cells. Then, the supernatant was transferred into a non-treated 96-well plate. The amount of cytokine release was quantified using Quantikine ELISA kit and Perforin Human ELISA Kit following manufacturer’s protocol. The data are presented as mean ± SD (n=3).

### Quantification of degranulation using surfaced CD107a (LAMP-1)

First, 10^4^ SKOV-3 cells were seeded in a 96-well plate and incubated overnight. The next day, a serial dilution of BiKE and trastuzumab ranging from 0 to 100 nM were added and incubated for 30 min at 37°C. Then, laNK92 (GFP^+^) or haNK92 was added at E:T ratio of 4 and the plate was incubated at 37°C for two hours. Next, Fc Blocker was added to the plate and incubated at 37˚C for additional one hour. Afterwards, APC-conjugated anti-CD107a antibody was added and incubated for one hour in an incubator. Finally, the plate was centrifuged down, and the supernatant was discarded. The cells were washed twice to remove excess antibodies and data was acquired by the Beckman Coulter CytoFLEX Cytometer. For data analysis, the GFP^+^ (laNK92) was gated to distinguish between effector and target cells. Next, the surfaced CD107a was quantified on GFP^+^ cell population.

## Results

### Expression of rCD16a and rHER2 and immunization of llama

rCD16a and rHER2 were genetically engineered and expressed in 293-F mammalian expression system, followed by purification. The SDS-PAGE analysis of purified proteins revealed the purity of both proteins to be >95% (**Supplementary Figures S2A, B**). The observed molecular weight for rCD16a is ~48 kDa, while the theoretical molecular weight (expected) is 21.83 kDa. The theoretical molecular weight of the HER2 is 72.63 kDa, while the migration of the protein on the gel showed a molecular weight close to 100 kDa. These differences indicate that the expressed rCD16a and rHER2 in mammalian cells went through post-translational modifications. According to existing literature, these two proteins have glycosylation sites, which make their actual molecular weights to be higher than their theoretical ones (15, 16). The purified rCD16a and rHER2 were then used to immunize llama. Comparison of IgG levels in the serum of llama before and after immunization showed a significant elevation of anti-CD16a and anti-HER2 serum antibodies against the proteins of interest, indicating a potent immune response (**Supplementary Figure S2C**).

### Isolation of high affinity and specificity anti-CD16a and anti-HER2 VHH clones

**Figure 1:**
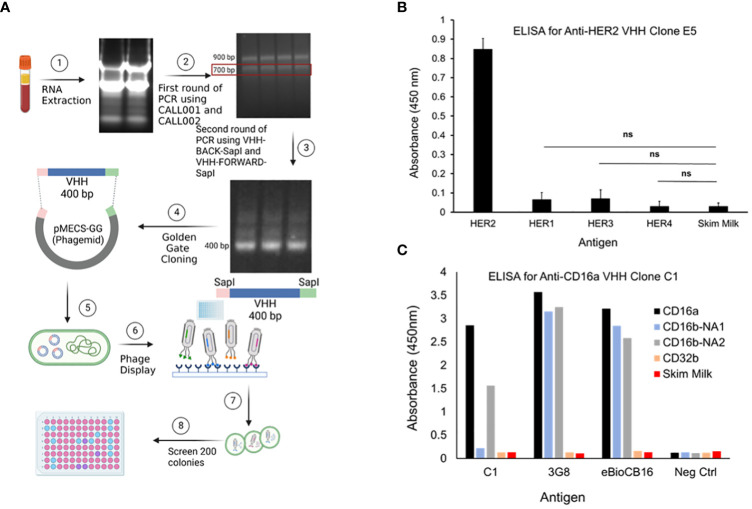
**(A)** PBMCs were isolated using Ficoll-Plaque method. Then, the RNAs were extracted, cDNA library was generated, genes amplified, and then cloned into phagemids. The phagemids were used in phage display and the screened candidates were used to infect TG1 bacteria. Colonies were selected, grown, lysed, and the lysates were screened by ELISA. **(B)** Evaluation of the selectivity of the top-performing anti-HER2 VHH clone E5 toward HER2 antigen. HER1, HER3, HER4, and skim milk were used as antigen controls. Data are presented as mean ± s.d. (n=3), *p<0.05, ns=not significant. **(C)** Evaluation of the selectivity of the top-performing anti-CD16a VHH clone C1 toward CD16a antigen. 3G8 and eBioCB16 anti-CD16a/b mAbs were used as antibody controls. CD16b-NA1, CD16b-NA2, and CD32b antigens were used as antigen controls.

Post immunization, llama blood was drawn and PBMCs were isolated, followed by cDNA and phagemid library generation, which were used in phage display. After four rounds of phage display, 190 colonies were selected, and lysed to obtain periplasmic extracts (**Figure 1A**). Lysates with the highest binding affinity toward CD16a and HER2 antigens were selected and examined for selectivity using ELISA. The two predominant alleles of CD16b, including human neutrophil antigen 1 (NA1) and NA2 (17), were used as controls. CD32b and skim milk were also used as antigen controls. 3G8 and eBioCB16 mAbs, which are anti-CD16a/b antibodies (detecting both CD16a and CD16b), were used as positive controls. The results of these experiments revealed anti-CD16a C1 VHH clone and anti-HER2 E5 VHH clone as top-performing constructs with high binding affinity and specificity/selectivity toward CD16a and HER2 antigens, respectively (**Figures 1B, C**). The E5 clone bound to HER2 antigen but did not interact with HER1, HER3, HER4, or skim milk. Therefore, the isolated E5 clone possesses both high specificity and selectivity. The C1 anti-CD16a clone did not interact with either CD32b or CD16b-NA1 antigens. However, the C1 clone was bound to the CD16b-NA2 antigen, although with less affinity. Considering the high sequence homology between CD16a and CD16b-NA2 (i.e., about 97% sequence identity and difference in four amino acid residues of the extracellular antibody-binding domains), the affinity of C1 clone toward CD16b-NA2 antigen was expected (17). Therefore, the isolated C1 clone possesses high specificity but not high selectivity.

### Evaluation of the selectivity of C1 and E5 VHH clones by flow cytometry

**Figure 2:**
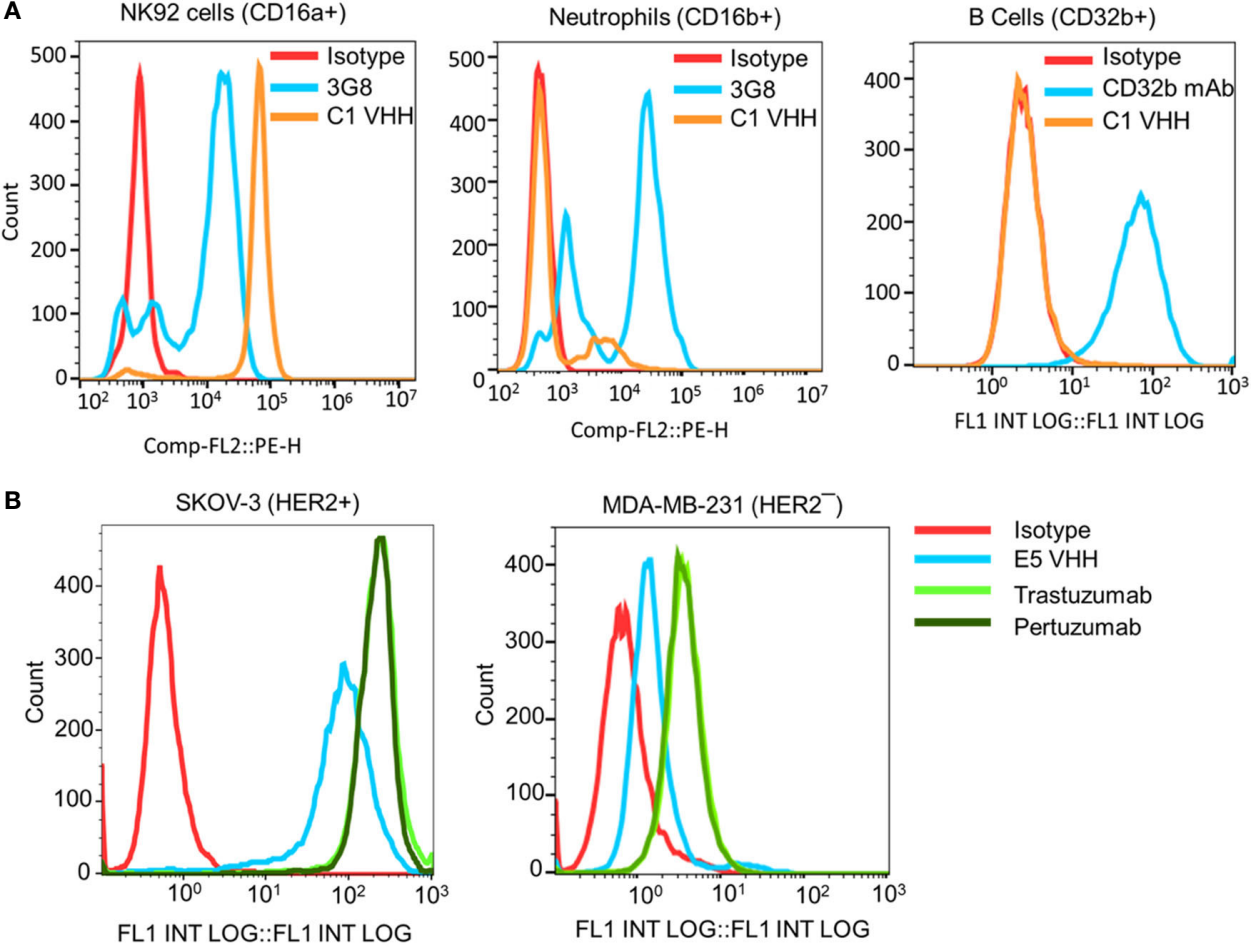
Evaluation of the selectivity of the C1 and E5 VHHs toward CD16a and HER2 antigens by flow cytometry. **(A)** Evaluation of the binding of anti-CD16a (C1 clone) VHH to NK92 cells (CD16a^+^), neutrophils (CD16b^+^), and B cells (CD32b^+^). 3G8 mAb against CD16a/b and AT10 mAb against CD32a/b were used as positive controls. **(B)** Evaluation of the binding of anti-HER2 (E5 clone) VHH to HER2^+^ SKOV-3 and HER2¯ MDA-MB-231 cancer cells. Trastuzumab and Pertuzumab were used as the positive controls. This figure shows the selectivity of C1 VHH toward NK cells (not neutrophils-NA1 or B cells) and E5 VHH toward HER2-positive cancer cells.

The selectivity of the C1 anti-CD16a VHH was evaluated using flow cytometry. In this experiment, NK92 cells (CD16a^+^), neutrophils (CD16b^+^/CD16a¯), and B cells (CD32b^+^/CD16a¯) were used as cell controls, whereas 3G8 mAb (anti-CD16a/b mAb) and AT10 (anti-CD32a/b mAb) were used as antibody controls. The results of this experiment showed that C1 VHH effectively bound to CD16a receptor on NK92 cells without cross-reactivity with neutrophils (NA1) and B cells (**Figure 2A**). In contrast, 3G8 mAb interacted with both NK92 and neutrophils indiscriminately (**Figure 2A** and **Supplementary Table S7**).

After C1 clone analysis, we evaluated the specificity/selectivity of the E5 anti-HER2 VHH using flow cytometry. For this purpose, equimolar amounts of purified VHHs, trastuzumab, and pertuzumab were used to detect the HER2 expression levels on the surface of SKOV-3 (HER2^+^) and MDA-MB-231 (HER2¯) cancer cells. The flow cytometry data showed that the selected E5 anti-HER2 VHH bound to HER2 on the surface of SKOV-3 cells but not MDA-MB-231 cells (**Figure 2B** and **Supplementary Table S8**). The level of VHHs binding to HER2, as determined by the percentage of labeled cells, was comparable to trastuzumab and pertuzumab (**Supplementary Table S8**).

### Construction of BiKE:HER2/CD16a and characterization

**Figure 3:**
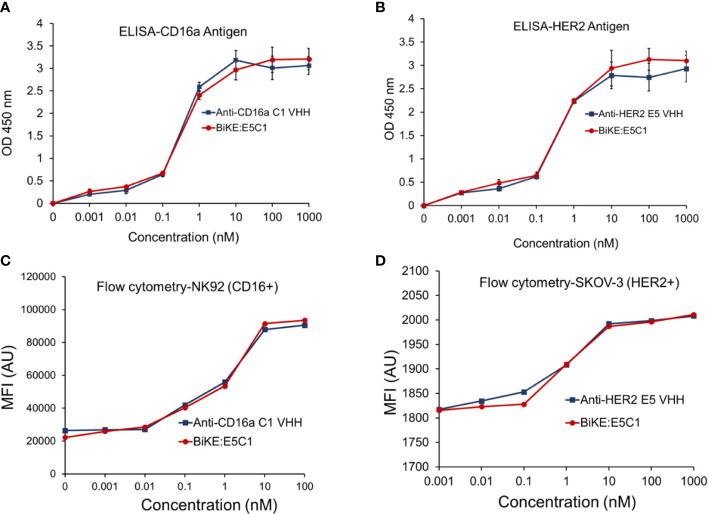
**(A)** Comparison of the binding of anti-CD16a C1 VHH with BiKE:E5C1 by ELISA. **(B)** Comparison of the binding of anti-HER2 E5 VHH with BiKE:E5C1 by ELISA. **(C)** Comparison of the binding of anti-CD16a C1 VHH with BiKE:E5C1 by flow cytometry using NK92 (CD16^+^) cells. **(D)** Comparison of the binding of anti-HER2 E5 VHH with BiKE:E5C1 by flow cytometry using SKOV-3 (HER2^+^) cells. This figure shows that the fusion of anti-CD16a VHH with anti-HER2 VHH did not affect their binding affinities toward the target antigens. Data are presented as mean ± s.d (n=3).

Using recombinant engineering, BiKE:HER2/CD16a was constructed by fusing C1 anti-CD16a VHH with E5 anti-HER2 VHH *via* a HMA semi-flexible linker (**Supplementary Table S9**). For simplicity, the construct will be shown as BiKE:E5C1. The SDS-PAGE data showed that the purified BiKE:E5C1 had above 95% purity, while the LC-MS graph showed the purified BiKE was free from any dimers or multimers (**Supplementary Figure S3**). The application of the HMA linker in the construction of bispecific antibodies has previously been demonstrated (18, 19). Then, the binding of the BiKE:E5C1 toward CD16a and HER2 antigens was evaluated by ELISA and flow cytometry. C1 anti-CD16a VHH and E5 anti-HER2 VHH were used as controls. Statistical analysis of the data (ELISA and flow cytometry) showed that the affinity of the BiKE:E5C1 toward CD16a and HER2 antigens remained intact and fusion of the two VHHs *via* the HMA linker did not negatively impact their bindings to the CD16a and HER2 antigens (t-test, *p*>0.05) (**Figures 3A-D**).

### Measurement of the binding affinities of VHHs and BiKE using BLI

**Figure 4:**
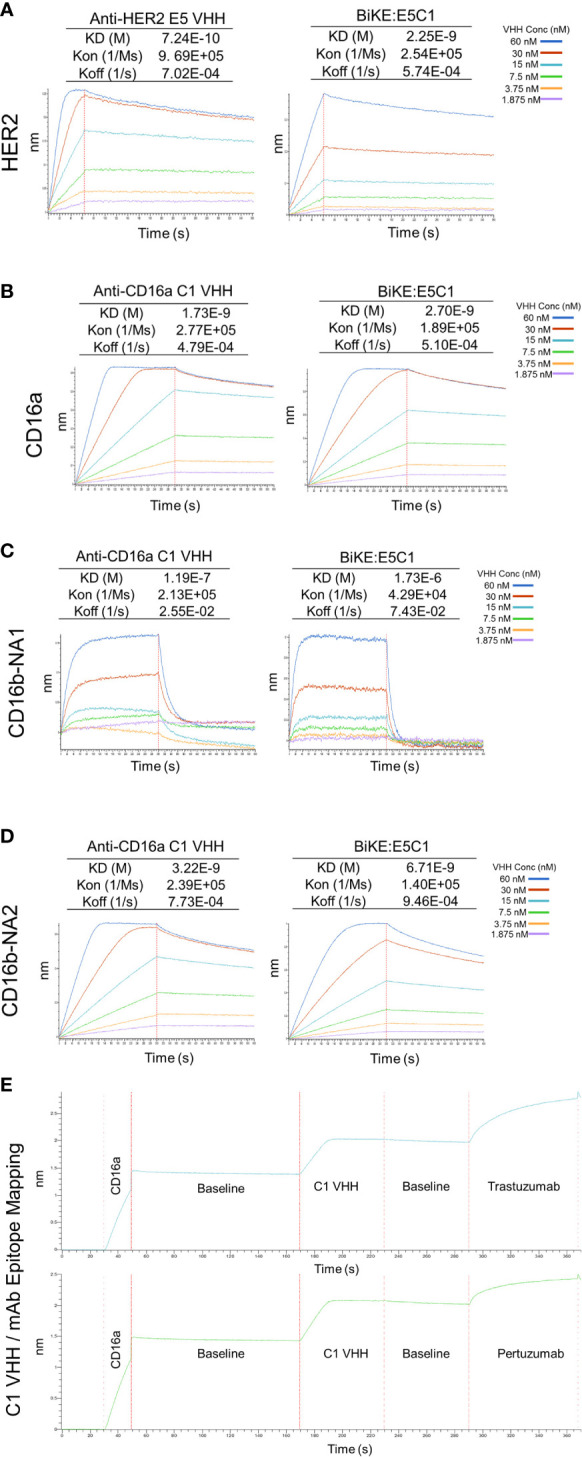
Measurement of K_D_ (binding constant), K_on_ (association constant), and K_off_ (dissociation constant) of VHHs and BiKE:E5C1 using BLI. **(A)** Measurment of the binding affinities (K_D_ values) of anti-HER2 VHH and BiKE:E5C1 toward HER2 antigen. **(B-D)** Measurment of the binding affinities (K_D_ values) of anti-CD16a VHH and BiKE:E5C1 toward CD16a, CD16b-NA1, and CD16b-NA2 antigens. **(E)** Epitope mapping for C1 VHH and mAbs (trastuzumab and pertuzumab) against CD16a antigen.

The affinity of the E5 and C1 VHHs, as well as BiKE:E5C1 toward their corresponding antigens, were quantified using BLI. First, the affinity of E5 VHH toward HER2 antigen was measured, then compared with affinity of BiKE:E5C1 toward HER2. The BLI sensograms showed the K_D_ values of 7.24 × 10^-10^ (M) and 2.25 × 10^-9^ (M) for E5 VHH and BiKE:E5C1, respectively (**Figure 4A**). While most antibodies have K_D_ values in the range of 10^-7^ to 10^-9^ (M), high-affinity antibodies have K_D_ values in the sub-nanomolar range (10^-9^ M). Therefore, both BiKE:E5C1 and E5 VHH are considered high-affinity antibodies.

Next, we measured the affinities of C1 VHH and BiKE:E5C1 toward CD16a, CD16b-NA1, and CD16-NA2 antigens. The BLI sensograms showed the affinities of 1.73 × 10^-9^ (M) and 2.7 × 10^-9^ (M) for C1 VHH and BiKE:E5C1 toward CD16a antigen, respectively (**Figure 4B**). The data also revealed that both C1 VHH and BiKE:E5C1 had low affinities (i.e., in the range of 10^-4^ to 10^-6^ M) toward CD16b-NA1, whereas they had high affinities toward CD16b-NA2 antigen (**Figures 4C, D**).

Furthermore, we examined whether the C1 anti-CD16a VHH binds to the same site as IgG1-based mAbs. For this purpose, we first treated CD16a antigen with C1 anti-CD16a VHH, followed by addition of IgG1-based mAbs such as trastuzumab and pertuzumab. This experiment showed that C1 VHH did not compete with trastuzumab and pertuzumab in binding to CD16a antigen, indicating the presence of different binding sites on CD16a antigen for these antibodies (**Figure 4E**).

### Measurement of ADCC and release of effector proteins using BiKE:E5C1, laNK92, and haNK92

**Figure 5:**
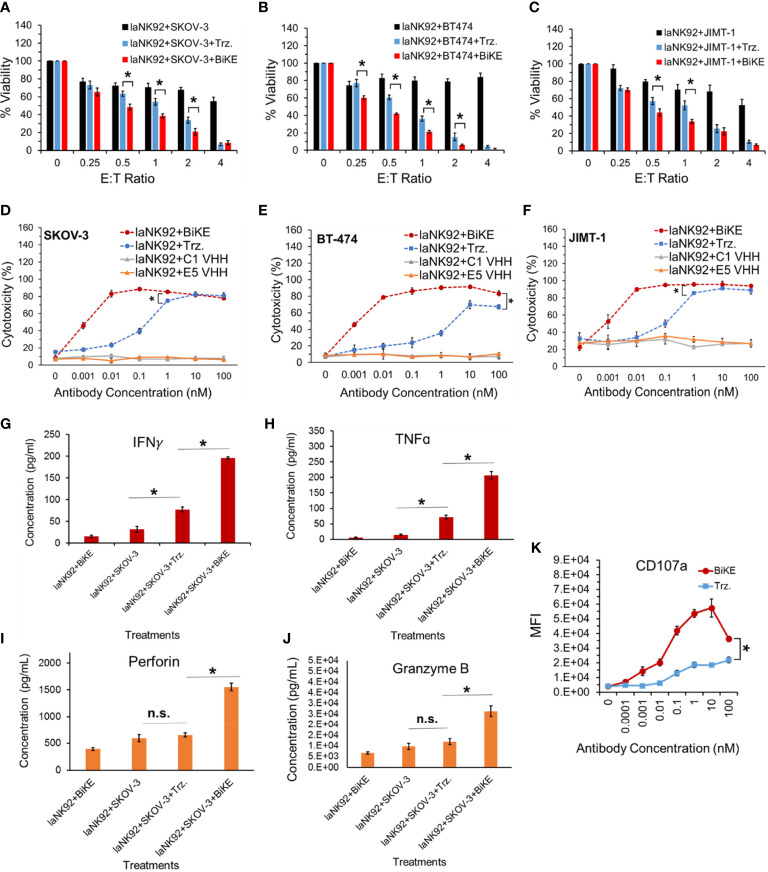
**(A-C)** Measurement of ADCC in three HER2^+^ cancer cell lines using laNK92 cells in combination with BiKE or Trastuzumab at different E:T ratios but fixed antibody concentration (100 nM). **(D-F)** Measurement of ADCC in three HER2^+^ cancer cell lines using laNK92 cells in combination with BiKE or Trastuzumab at different antibody concentrations but fixed E:T ratio of 4. **(G-J)** Measurement of IFN-γ, TNF-α, Perforin, and Granzyme B after incubation of SKOV-3 cells with laNK92 cells in the presence of BiKE or Trastuzumab (Trz.) using ELISA. **(K)** Measurement of change in CD107a expression (laNK92 degranulation) at different antibody concentrations using flow cytometry. The data are shown as mean ± s.d. (*t-test, p<0.05, n.s.: not significant).

To determine whether BiKE:E5C1 provides an advantage in terms of ADCC over the currently available best-in-class anti-HER2 mAb (i.e., trastuzumab), a cell toxicity assay was performed. As effector cells, both laNK92 (F176) and haNK92 (V176) cells were used. It has been shown that the CD16a (V176) has a relatively higher affinity toward the Fc region of mAbs (20). HER2^+^ cancer cell lines SKOV-3, BT474, and JIMT-1 were seeded under adherent conditions and used as target cells. First, cancer cells were treated with laNK92 cells alone, laNK92 plus trastuzumab (equivalent of 100 nM), and laNK92 plus BiKE:E5C1 (equivalent of 100 nM) followed by measurement of cell viability. The results of this experiment showed that trastuzumab significantly increased cytotoxicity of laNK92 cells at most E:T ratios; however, in all three tested cell lines, the ADCC of BiKE:E5C1 was superior to trastuzumab (**Figures 5A–C**). Maintaining the E:T ratio of 4 at which both trastuzumab and BiKE:E5C1 could kill more than 90% of cancer cells, we measured the ADCC using antibodies of different concentrations. The results of this experiment showed that BiKE:E5C1 was approximately 100-fold more potent than trastuzumab (**Figures 5D–F**). To evaluate whether the death of cancer cells was due to stimulation of the laNK92 cells by BiKE, we measured the concentrations of cytotoxic proteins and cytokines, including Perforin, Granzyme B, IFN-γ, and TNF-α during the ADCC experiment. Herein, we used SKOV-3 cells as target cells since our data along with previous literature have shown that SKOV-3 cells have limited expression of NKG2D ligands (i.e., MICA/B) on their surfaces (**Supplementary Figure S4**) (21). As a result, the probability of laNK92 cell stimulation through NKG2D ligands on SKOV-3 cells (in the absence of BiKE) is significantly reduced. The results of this experiment showed that BiKE played a significant role in stimulating laNK92 cells to release the cytotoxic proteins and cytokines at rates substantially higher than trastuzumab (**Figures 5G-K**). The higher rate of laNK92 stimulation with BiKE:E5C1 explains the observed higher rate of ADCC in cancer cells, which were treated with laNK92 plus BiKE:E5C1 compared to those treated with laNK92 plus trastuzumab. As expected, the data also showed that incubation of laNK92 cells with SKOV-3 (without BiKE) had a limited effect on the release of cytotoxic proteins and cytokines (**Figures 5G–K**). During the short two hours of co-incubation, we did not observe a significant release of perforin and granzyme B from laNK92 cells that were incubated with SKOV-3 and trastuzumab (**Figures 5G–J**). This can be attributed to low-affinity interaction between Fc region and CD16a and short incubation time. In contrast, we did observe a significant release of IFN-γ and TNF-α from laNK92 cells that were incubated with SKOV-3 and trastuzumab for 24 hours.

**Figure 6:**
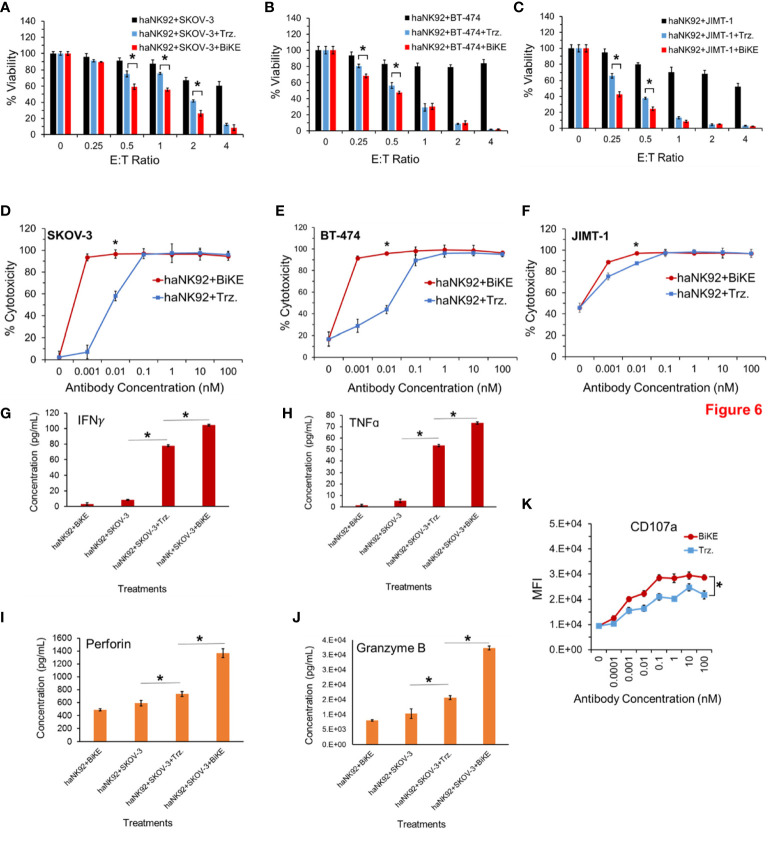
**(A-C)** Measurement of ADCC in HER2^+^ cancer cell lines using haNK92 cells in combination with BiKE or Trastuzumab at different E:T ratios but fixed antibody concentration (100 nM). **(D-F)** Measurement of ADCC in HER2^+^ cancer cell lines using haNK92 cells in combination with BiKE or trastuzumab at different antibody concentrations but fixed E:T ratio of 4. **(G-J)** Measurement of IFN-γ, TNF-α, Perforin, and Granzyme B after incubation of SKOV-3 cells with haNK92 cells in the presence of BiKE or trastuzumab (Trz.) using ELISA. **(K)** Measurement of change in CD107a expression (haNK92 degranulation) at different antibody concentrations using flow cytometry. The data are shown as mean ± s.d. (*t-test, p<0.05).

To determine whether BiKE:E5C1 provides an advantage in terms of ADCC over trastuzumab in patients with high affinity NK (V176) cells, a cell toxicity assay using haNK92 (V176) was performed. HER2^+^ cancer cells were seeded in adherent condition as mentioned above and treated with haNK92 cells alone, haNK92 plus trastuzumab (equivalent of 100 nM), or haNK92 plus BiKE:E5C1 (equivalent of 100 nM), followed by cell viability measurement. Cancer cells were also treated under the same conditions at a fixed E:T ratio using different concentrations of antibodies. The results of these experiments showed that ADCC with BiKE:E5C1 was superior to trastuzumab not only at different E:T ratios (**Figures 6A–C**), but also at different concentrations (**Figures 6D–F**). Measurement of the release of effector proteins also showed that BiKE:E5C1 activates haNK92 cells significantly more than trastuzumab (**Figures 6G–K**). Due to the high-affinity interaction between Fc region and CD16a, regardless of incubation time, the release of effector proteins from haNK92 cells, which were incubated with SKOV-3 cells plus trastuzumab, was significantly higher than haNK92 cells without trastuzumab.

### Evaluation of the ability of BiKE and haNK92 to kill HER2^+^ cancer cells in suspension

**Figure 7:**
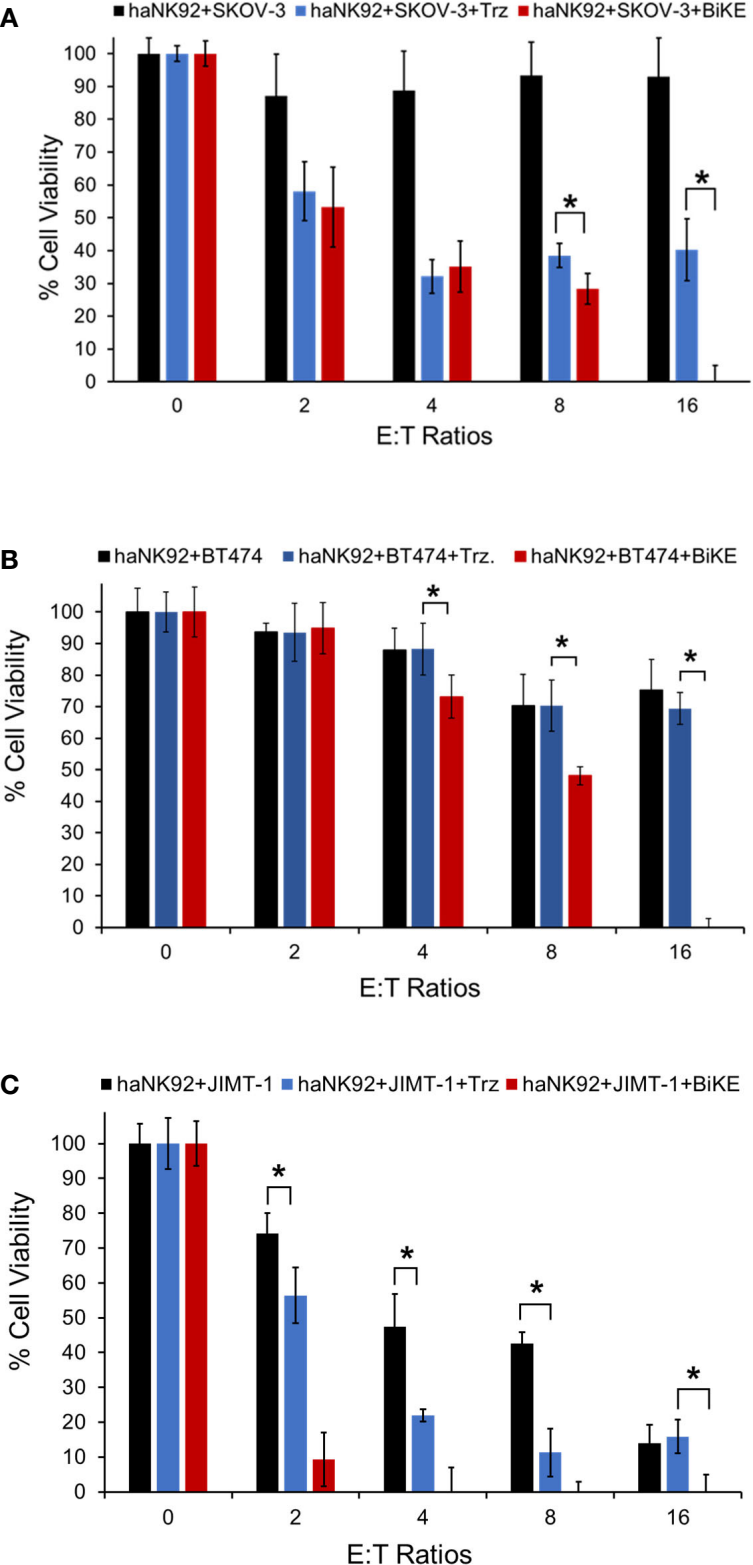
Evaluation of the ADCC for BiKE:E5C1 by using a cell toxicity assay. **(A-C)** SKOV-3, BT474, and JIMT-1 cells were incubated with haNK92 cells for 4 hours in suspension at different E:T ratios. ADCC was measured after treating cancer cells with trastuzumab (Trz., 100 nM) or BiKE:E5C1 (100 nM). This figure shows that BiKE:E5C1 induced significantly higher ADCC in comparison to Trz. The data are presented as mean± s.d. (*t-test, *p*<0.05).

To determine whether BiKE:E5C1 can facilitate the recognition and killing of the HER2^+^ cancer cells in suspension, representing circulating cancer cells, by haNK92 cells, an ADCC assay under non-adherent conditions was performed. In this experiment, both cancer cells and haNK92 cells were seeded in non-adherent plates, followed by the addition of either BiKE:E5C1 or trastuzuamb. The results of this study showed BiKE:E5C1 assisted in the killing of HER2^+^ cancer cells by haNK92 cells more effectively than trastuzumab (**Figures 7A–C**).

## Discussion

IgG-based antibodies mediate the clearance of cancer cells *via* the engagement of CD16a receptors on natural killer cells and macrophages by eliciting ADCC and antibody-dependent cell phagocytosis (ADCP) (22, 23). For example, Lee et. al., have shown that infusion of CD16a-positive NK cells potentiates the anticancer activity of trastuzumab in patients with refractory HER2-positive solid tumors (24). However, the affinity of mAb Fc region toward CD16a is weak (K_D_ of ~8 × 10^-7^) with cross-reactivity with CD16b and CD32b receptors (25). As a result, the therapeutic efficacy of the current commercially available mAbs has significant room for improvement and there is a need for development of antibodies that can overcome the affinity and cross-reactivity shortcomings. To fill this gap, we immunized llama with rCD16a and rHER2 to generate VHHs against the antigens followed by identification and isolation of C1 anti-CD16a VHH clone and E5 anti-HER2 VHH clone. VHH, with the small size of 15 kDa, lacks light chains and Fc region, but possesses high target affinity and stability, and can be easily produced in both eukaryotic and prokaryotic cells (11, 26). Additionally, VHHs have a low risk of immunogenicity due to their small sizes and a high degree of homology between their encoding genes and the ones encoding VH3 family of human antibodies (27, 28).

To isolate clones with high affinity and selectivity, we first performed ELISA, which showed that the C1 and E5 VHHs were highly bound and specific toward CD16a and HER2 antigens, respectively (**Figure 1**). The isolated C1 VHH also showed strong binding to NK92 (CD16^+^) cells with negligible binding to neutrophils and B cells (**Figure 2A**). Since C1 VHH binds effectively to NK cells without being drained by neutrophils or inhibiting the activation of B cells, it could potentially produce a significantly better therapeutic response in cancer patients (5–7).

To create BiKE for the treatment of HER2^+^ cancer cells, we recombinantly fused the C1 VHH with E5 VHH *via* an HMA (semi-rigid linker) to generate BiKE:E5C1. Several studies have demonstrated that less flexible linkers yield higher agonistic activity (29). For instance, using small-angle x-ray scattering and ensemble modeling, Orr et al. illustrated that monoclonal antibodies with less flexible hinge can adopt fewer possible conformations compared to flexible ones, thus eliciting higher agonistic activity (29, 30). Using ELISA, flow cytometry, and BLI we examined whether the engineered BiKE:E5C1 maintained its binding affinity and selectivity toward its corresponding antigens. The results of these studies showed that the binding affinity of BiKE:E5C1 toward CD16a and HER2 antigens were similar to its parent VHHs (**Figures 3**, **4**). Our BLI data showed that BiKE:E5C1 is specifically bound to CD16a and CD16b-NA2 but not CD16b-NA1 (**Figures 4B–D**). Recently, it was found that neutrophils destroy antibody-coated cancer cells by a mechanism, termed trogoptosis. In this approach, neutrophils take up small pieces of the cancer cell membrane leading to mechanical injury of cancer cells and ultimately cell death (31). Therefore, patients with CD16b-NA2 allele are expected to be responsive to BiKE-induced ADCC, perhaps not as strongly as those with CD16b-NA1 allele. In clinical trials, patients with CD16b-NA1 allele can be screened to become primary candidates for BiKE:E5C1 therapy, whereas those with CD16b-NA2 allele could be recruited in another arm to examine their level of responsiveness in comparison to CD16b-NA1 allele and control groups. Importantly, the epitope mapping experiment by BLI showed that the C1 VHH recognizes a different epitope on CD16a receptor than IgG1-based mAbs trastuzumab and pertuzumab. This outcome has two significant implications. First, the generated BiKE:E5C1 could be used with other IgG1-based mAbs (e.g., PD-L1 inhibitors) for combination therapy since they don’t compete with each other in binding to CD16a receptors. Second, it suggests that the IgG1 in the plasma may not interfere with the binding of BiKE:E5C1 to CD16a antigen on NK cells.

We then examined the ADCC of Trastuzumab and compared it with BiKE:E5C1 using laNK92 and haNK92 cells. Approximately 10% of humans are homozygous for the higher affinity CD16a (V176/V176) allele, whereas the rest of the human population are either low affinity homozygous (F176/F176) or heterozygous (F176/V176) (20). The binding affinities of CD16a (V176) and CD16a (F176) receptors toward IgG1 Fc region have been reported to be 20×10^5^ and 11.7×10^5^ (M^-1^), respectively (25). To be consistent with literature, these binding affinities can also be shown as 5×10^-7^ and 8×10^-7^ (M), respectively. Since clinical data have shown that patients with CD16a (V176) allele responded better to antibody therapies than those with CD16a (F176) allele (32), several groups are making efforts to engineer new generation of mAbs with higher affinity toward CD16a receptors. For example, margetuximab is an Fc-engineered anti-HER2 monoclonal antibody with higher affinity toward CD16a. Margetuximab’s affinity toward CD16a (F176) is measured to be ~99×10^-9^ (M), which is a ten-fold improvement over IgG1 (33). However, the Fc-engineering also modestly increased margetuximab’s affinity toward CD32b inhibitory receptor (~2-folds). Phase 3 clinical data have shown that patients with CD16a (F176) allele who received margetuximab plus chemotherapy responded better than those who received trastuzumab plus chemotherapy. Though this difference in outcomes was not observed in CD16a (V176) patients (4). Considering the affinity of BiKE:E5C1 toward CD16a (F176), which is 2.7×10^-9^ (M) and 36-fold higher than margetuximab, we expect to observe better therapeutic response with BiKE:E5C1 than margetuximab in future clinical trials.

The results of our experiments with both laNK92 and haNK92 showed that BiKE provides superior ADCC than trastuzumab regardless of the NK92 cell subtype (**Figures 5**, **6**). It was also notable that BiKE was ~100 fold more potent than trastuzumab meaning that it could kill HER2^+^ cancer cells with concentrations at least 100 times less than trastuzumab (**Figures 5D–F**, **6D–F**). It has previously been shown that JIMT-1 cells are resistant to trastuzumab. Interestingly, prior reports (34, 35), and our data show that JIMT-1 cells are not resistant to ADCC by trastuzumab plus NK cells. Therefore, tumors that are resistant to monotherapy with trastuzumab, could be a candidate for BiKE/NK combination therapy. Our experiments also demonstrated that the superior ADCC with BiKE over trastuzumab is directly correlated to higher production of cytokines (TNF-α and IFN-γ) and pore-forming peptides (Granzyme B and Perforin) (**Figures 5**, **6**). Published data by others also show that the release of Granzyme B, Perforin, TNF-α, and IFN-γ is essential for NK cell anticancer activity (36). In comparison to SKOV-3 cells, it was also apparent that JIMT-1 cells were more sensitive to not only NK92 cells, but also NK92 plus antibodies (**Figures 6D–F**). The observed higher level of vulnerability to NK92 cells can be attributed to higher expression levels of NKG2D ligands on the surface of JIMT-1 in comparison to SKOV-3 cells making JIMT-1 cells a better target for NK92 cells. We also examined whether BiKE could facilitate recognition of cancer cells under non-adherent conditions (in suspension). The rationale behind this experiment was to evaluate the ability of the BiKE to augment the cytotoxicity of NK cells against circulating tumor cells. For this purpose, we used haNK92 in combination with trastuzumab or BiKE to induce ADCC in HER2^+^ cancer cells. Overall, the combined cell toxicity data of the three cell lines showed that BiKE:E5C1 induced significantly higher toxicity to cancer cells compared to trastuzumab (**Figure 7**). This outcome could be explained by the fact that the binding affinity of BiKE:E5C1 toward CD16a is at least 100 times higher than mAbs.

In parallel to our work, other groups are also making efforts to create BiKEs with higher affinity and specificity/selectivity toward CD16a on NK cells to elicit a better therapeutic response in cancer patients (9). For instance, Affimed Therapeutics has developed a high affinity/selectivity IgG-based anti-CD16a antibody (known as ROCK^®^ platform), which is in Phase I clinical studies for the treatment of lymphomas (NCT04101331) (37). In general, VHH-based BiKEs provide significant advantages over heavily engineered IgG-based BiKEs in different ways, including ease of production, higher stability, lack of complexity, and meaningfully smaller size. The smaller size of VHH-based BiKEs in combination with their high affinity towards HER2 provides better tumor penetration and retention capabilities, potentially yielding higher anticancer activity. While the freely circulating VHH-based BiKEs are expected to have faster rate of clearance from the blood than IgG-based mAbs, BiKEs that are bound to NK cells are expected to linger longer due to high affinity interactions. Whether this faster clearance of unbound BiKEs negatively affects their therapeutic efficacy or positively their safety profile, is something that can be determined in clinical trials.

## Conclusions

Our data show that the engineered BiKE:E5C1 has high affinity and specificity toward CD16a with negligible cross-reactivity with CD16b-NA1 and CD32b. Upon activation of CD16^+^ NK92 cells, the engineered BiKE:E5C1 induced the release of higher amounts of Perforin, Granzyme B, TNF-α, and IFN-γ, in comparison to trastuzumab leading to higher ADCC with BiKE:E5C1. Since BiKE:E5C1 recognizes a different epitope on CD16a antigen than trastuzumab, the opportunity for combination therapy with other antibody drugs such as checkpoint inhibitors and antibody-drug conjugates arises. Overall, the data demonstrate the creation of a new BiKE with the potential to elicit a superior therapeutic response in patients with HER2^+^ cancer than existing anti-HER2 mAbs.

## Data availability statement

The original contributions presented in the study are included in the article/**Supplementary Material**. Further inquiries can be directed to the corresponding author.

## Author contributions

AH: Conceptualization, Methodology, Validation, Formal analysis, Resources, Writing - Review & Editing, Visualization, Supervision, Project administration, Funding acquisition. SN: Methodology, Validation, Formal analysis, Investigation, Writing - Original Draft, Visualization. GL: Methodology. SE: Investigation. GY: Methodology, Investigation. VV: Methodology, Investigation. All authors contributed to the article and approved the submitted version.
