# Supplementary material for: Bispecific killer cell engager with high affinity and specificity toward CD16a on NK cells for cancer immunotherapy
Source: Front Immunol. 2023 Jan 6;13:1039969. doi: 10.3389/fimmu.2022.1039969 (PMC9852913; doi:10.3389/fimmu.2022.1039969)
Supplement: Supplementary file 1 [file DataSheet_1.pdf]

Supplementary Materials

Engineering of Bispecific Killer Cell Engager with High Affinity and Specificity toward CD16a on NK Cells and HER2 on Cancer Cells for Cancer Immunotherapy

Shahryar Khoshtinat Nikkhoi[1], Geng Li[1], Suha Eleya[1], Ge Yang[1], Venu Gopal Vandavasi[3], and *Arash Hatefi[1,2]

[1]Department of Pharmaceutics, Rutgers University, Piscataway, New Jersey 08854, USA

[2]Cancer Pharmacology Program, Cancer Institute of New Jersey, Rutgers University, New Brunswick, NJ 08901, USA

[3]Department of Chemistry, Biophysics Core Facility, Princeton University, Princeton, New Jersey, 08544, USA

Supplementary Tables

Supplementary Table 1: The list of the materials used to generate, isolate, and characterize anti-CD16a VHH, anti-HER2 VHH, and BiKE:HER2/CD16a .

Item	Vendor	Catalogue No.
0.2 µm Syringe filters with acrylic housing	VWR (PA, USA)	28145-501
1-Step™ Turbo TMB-ELISA Substrate Solution	Thermo Scientific™ (MA, USA)	34022
2-Mercaptoethanol	Gibco™ (NY, USA)	21985023
2x YT media	Sigma-Aldrich (MA, USA)	Y2377-250G
Abgene™ 96 Well Deepwell Storage Plate	Thermo Scientific™ (MA, USA)	AB0932
Acetic acid (17.48 M)	Sigma-Aldrich (MA, USA)	A6283-100ML
AEBSF Protease Inhibitor	Thermo Scientific™ (MA, USA)	78431
alamarBlue™ HS Cell Viability Reagent	Thermo Scientific™ (MA, USA)	A50100
Animal-Free Recombinant Human IL-2	Peprotech (NJ, USA)	AF-200-02-250UG
Anti-CD32B + CD32A antibody [AT10]	Abcam (MA, USA)	ab41899
Anti-Clumping Agent (1000X)	Gibco™ (NY, USA)	0010057AE
Anti-HA tag antibody (HRP)	Abcam (MA, USA)	ab1190
B Lymphoblastoid Cell lines	Charles River (CA, USA)	B-LCL
Biotinylated Human Fc gamma RIIIA / CD16a (F176) Protein, Avitag™, His Tag (SPR & BLI verified)	Acrobiosystems (DE, USA)	CDA-H82E8-25ug
Biotinylated Human Fc gamma RIIIB / CD16b (NA1) Protein, His,Avitag™ (SPR & BLI verified)	Acrobiosystems (DE, USA)	CDB-H82E4-25ug
Biotinylated Human Fc gamma RIIIB / CD16b (NA2) Protein, His,Avitag™ (SPR & BLI verified)	Acrobiosystems (DE, USA)	CDB-H82Ea-25ug
Biotinylated Human Her2 / ErbB2 Protein, His,Avitag™	AcroBiosystem (DE, USA)	HE2-H82E2-25ug
Bovine Albumin Fraction V (7.5% solution)	Gibco™ (NY, USA)	15260037
BT-474 breast cancer cells	ATCC (VA, USA)	HTB-20™
Carbenicillin (Disodium)	GoldBio (MO, USA)	C-103-25

CD16 Monoclonal Antibody (3G8), Functional Grade, eBioscience™	Thermo Scientific™ (MA, USA)	16-0166-82
CD16 Monoclonal Antibody (eBioCB16 (CB16)), eBioscience™	Thermo Scientific™ (MA, USA)	14-0168-82
D-(+)-Glucose	MiliporeSigma (MA, USA)	G8270-1KG
Deoxynucleotide (dNTP) Solution Mix	NEB (MA, USA)	N0447S
DMEM/F-12, GlutaMAX™ supplement	Gibco™ (NY, USA)	10565018
DPBS, no calcium, no magnesium	Thermo Scientific™ (MA, USA)	14190250
DPBS, no calcium, no magnesium	Gibco™ (NY, USA)	14190144
ELISA Coating Buffer	Bio-Rad (PA, USA)	BUF030C
Escherichia coli (Migula) WK6	ATCC (VA, USA)	ATCC 47078
Ethylenediaminetetraacetic acid disodium salt dihydrate	Sigma-Aldrich (MA, USA)	E5134-100G
Fetal Bovine Serum, qualified, United States	Gibco™ (NY, USA)	26140079
Fetal Bovine Serum, qualified, United States	Gibco™ (NY, USA)	26140079
Folic Acid	Sigma-Aldrich (MA, USA)	F8758-5G
FreeStyle™ 293 Expression Medium	Gibco™ (NY, USA)	12338018
FreeStyle™ 293-F Cells	Gibco™ (NY, USA)	R79007
Gel Loading Dye, Purple	NEB (MA, USA)	B7024S
Gene Pulser®/MicroPulser™ Electroporation Cuvettes, 0.1 cm gap	Bio-Rad (PA, USA)	1652083
Goat Anti-Rabbit IgG H&L (FITC)	Abcam (MA, USA)	ab6717
Histag Polyclonal Antibody, DyLight 680	Thermo Scientific™ (MA, USA)	600-444-382
Histopaque®-1077 (Ficoll)	Sigma-Aldrich (MA, USA)	10771-500ML
Horse Serum, New Zealand origin	Gibco™ (NY, USA)	16050122
Human Granzyme B Quantikine ELISA Kit	R&D Systems (MN, USA)	DGZB00
Human IFN-gamma Quantikine ELISA Kit	R&D Systems (MN, USA)	DIF50C
Human LAMP-1/CD107a APC-conjugated Antibody	R&D Systems (MN, USA)	IC4800A
Human TNF-alpha Quantikine ELISA Kit	R&D Systems (MN, USA)	DTA00D

Human TruStain FcX™ (Fc Receptor Blocking Solution)	BioLegend (CA, USA)	422301
Hybri-Care Medium	ATCC (VA, USA)	46-X
Hydrochloric acid solution	Sigma-Aldrich (MA, USA)	H9892-100ML
Insulin (10 mg/mL)	Sigma-Aldrich (MA, USA)	I0516-5ML
Isopropyl β-d-1-thiogalactopyranoside (IPTG)	Teknova (CA, USA)	I3325
JIMT-1	Gift from Dr. Jason S. Lewis (MSKCC, NY)	N/A
LB Miller Broth	IBI Scientific (IA, USA)	IB49030
Llama IgG-heavy and light chain Antibody	Bethyl (TX, USA)	A160-100P
McCoy's 5A (Modified) Medium	Gibco™ (NY, USA)	16600108
MEM α, no nucleosides	Gibco™ (NY, USA)	12561056
MicroAmp™ Fast Reaction Tube with Cap	Thermo Scientific™ (MA, USA)	4358297
Milk powder, non-fat (skimmed milk), Powder	VWR (PA, USA)	97063-958
Miller's LB Agar	IBI Scientific (IA, USA)	IB49100
myo-Inositol	Sigma-Aldrich (MA, USA)	I7508-100G
Natural killer (NK-92®) cell line (low affinity; 176F)	ATCC (VA, USA)	PTA-8837™
Natural Killer cell Line No-GFP-CD16.NK-92® cell Line (high affinity; 176V)	ATCC (VA, USA)	PTA-6967™
Neutrophils	HemaCare (CA, USA)	PB011C-1
Ni-NTA Agarose	Qiagen (MD, USA)	30230
Nuclease-Free Water (not DEPC-Treated)	Thermo Scientific™ (MA, USA)	AM9937
Nunc MaxiSorp™ flat-bottom	Invitrogen™ (MA, USA)	44-2404-21
Nunc™ Lab-Tek™ II Chamber Slide™ System	Thermo Scientific™ (MA, USA)	154526PK
Octet® Streptavidin (SA) Biosensor	Sartorius (PA, USA)	18-5019
Opti-MEM™ I Reduced Serum Medium	Thermo Scientific™ (MA, USA)	31985062
Penicillin-Streptomycin (10,000 U/mL)	Gibco™ (NY, USA)	15140122
Penicillin-Streptomycin (10,000 U/mL)	Gibco™ (NY, USA)	15140122

Perforin Human ELISA Kit	Thermo Scientific™ (MA, USA)	BMS2306
Pertuzumab (Perjeta TM, Genentech)	Cancer Institute of New Jersey Pharmacy Store	N/A
pET-28a-c(+) vectors	Novagen (MA, USA)	69864-3
Pierce™ BCA Protein Assay Kit	Thermo Scientific™ (MA, USA)	23225
Platinum™ II Hot-Start Green PCR Master Mix	Thermo Scientific™ (MA, USA)	14001012
Polyethylene glycol	Sigma-Aldrich (MA, USA)	81260-1KG
Polyethylenimine, Linear, MW 25000	PolyScience (PA, USA)	23966-1
Precision Plus Protein™ Dual Color Standards	Bio-Rad (PA, USA)	1610374
Protein LoBind tubes, PCR clean	Eppendorf (MA, USA)	0030108302
Q5® High-Fidelity DNA Polymerase	NEB (MA, USA)	M0491S
Q5® High-Fidelity PCR Kit	NEB (MA, USA)	E0555S
QIAGEN Plasmid Mega Kit	Qiagen (MD, USA)	12181
QIAprep Spin Miniprep Kit	Qiagen (MD, USA)	27106
QIAquick Gel Extraction Kit	Qiagen (MD, USA)	28704
QIAquick PCR Purification Kit	Qiagen (MD, USA)	28106
Quick-Load® Purple 100 bp DNA Ladder	NEB (MA, USA)	N0551S
RIPA Lysis and Extraction Buffer	Thermo Scientific™ (MA, USA)	89901
RNase-Free DNase Set	Qiagen (MD, USA)	79254
RNeasy Mini Kit	Qiagen (MD, USA)	74104
RPMI-1640 Medium	Sigma-Aldrich (MA, USA)	R8758-6X500ML
S.O.C. Medium	Invitrogen™ (MA, USA)	15544034
SapI restriction enzyme	NEB (MA, USA)	R0569L
SK-BR-3 breast cancer cells	ATCC (VA, USA)	HTB-30™
SKOV-3 ovarian cancer cells	ATCC (VA, USA)	HTB-77™
Sucrose	Sigma-Aldrich (MA, USA)	S7903-1KG
SuperScript™ IV First-Strand Synthesis System	Thermo Scientific™ (MA, USA)	18091050

SYBR™ Safe DNA Gel Stain	Thermo Scientific™ (MA, USA)	S33102
T4 DNA Ligase	NEB (MA, USA)	M0202L
Terrific Broth	IBI Scientific (IA, USA)	IB49141
TG1 Electrocompetent Cells	Lucigen (WI, USA)	60502-2
TrackIt™ 100 bp DNA Ladder	Thermo Scientific™ (MA, USA)	10488058
Trastuzumab (Trazimera TM, Pfizer)	Cancer Institute of New Jersey Pharmacy Store	N/A
Trypan Blue Solution, 0.4%	Gibco™ (NY, USA)	15250061
Trypsin-EDTA solution	MiliporeSigma (MA, USA)	T4049-100ML
UltraPure™ Agarose	Thermo Scientific™ (MA, USA)	16500100
UltraPure™ DNA Typing Grade™ TAE Buffer	Thermo Scientific™ (MA, USA)	24710030
VCSM13 Interference-Resistant Helper Phage	Integrated Science (VA, USA)	200251
Ventilating Adhesive Plate Seals	Thermo Scientific™ (MA, USA)	AB0718
WELQut Protease (5 U/µL)	Thermo Scientific™ (MA, USA)	EO0861

Supplementary Table 2: The amino acid sequence of the secretory rCD16 protein with a theoretical molecular weight of 24.56 kDa

Name	Amino Acid Sequence
rCD16a (Ectodomain)	**METDTLLLWVLLLWVPGSTGD**AAQPARRASL<u>WELQ</u>GMRTEDLPKAVVFL EPQWYRVLEKDSVTLKCQGAYSPEDNSTQWFHNESLISSQASSYFIDAATVD DSGEYRCQTNLSTLSDPVQLEVHIGWLLLQAPRWVFKEEDPIHLRCHSWKNT ALHKVTYLQNGKGRKYFHHNSDFYIPKATLKDSGSYFCRGLFGSKNVSSETV NITITQGLAVSTISSFFPPGYQ<u>WELQ</u>LEEGP*HHHHHHHHHHHH***

***Secretory signal** – rCD16a ectodomain - <u>Protease site</u> - *Histag*

Supplementary Table 3: The amino acid sequence of the secretory rHER2 protein with theoretical molecular weight of 73.56 kDa

Name	Amino Acid Sequence
rHER2 Extracellular Domain	**METDTLLLWVLLLWVPGSTGD**AAQPARRASLWELQTQVCTGTDMKLRLPA<br>SPETHLDMLRHLYQGCQVVQGNLELTYLPTNASLSFLQDIQEVQGYVLIAHNQ<br>VRQVPLQRLRIVRGTQLFEDNYALAVLDNGDPLNNTTPVTGASPGGLRELQLR<br>SLTEILKGGVLIQRNPQLCYQDTILWKDIFHKNNQLALTLIDTNRSRACHPCSP<br>MCKGSRCWGESSEDCQSLTRTVCAGGCARCKGPLPTDCCHEQCAAGCTGPKH<br>SDCLACLHFNHSGICELHCPALVTYNTDTFESMPNPEGRYTFGASCVTACPYN<br>YLSTDVGSCTLVCPLHNQEVTAEDGTQRCEKCSKPCARVCYGLGMEHLREVR<br>AVTSANIQEFAGCKKIFGSLAFLPESFDGDPASNTAPLQPEQLQVFETLEEITGY<br>LYISAWPDSLPDLSVFQNLQVIRGRILHNGAYSLTLQGLGISWLGLRSLRELGS<br>GLALIHHNTHLCFVHTVPWDQLFRNPHQALLHTANRPEDECVGEGLACHQLC<br>ARGHCWGPGPTQCVNCSQFLRGQECVEECRVLQGLPREYVNARHCLPCHPEC<br>QPQNGSVTCFGPEADQCVACAHYKDPPFCVARCPSGVKPDLSYMPIWKFPDEE<br>GACQPCPINCTHSCVDLDDKGCPAEQRASPLTWELQLEEGP*HHHHHHHHHH*<br>*H**

***Secretory signal** - Protease site - HER2 ectodomain - *Histag*

Supplementary Table 4: The list of primers and the corresponding sequences used to make VHH cDNAs.

**Name**	**Sequence**
CALL001	5′- GTCCTGGCTGCTCTTCTACAAGG-3′
CALL002	5′- GGTACGTGCTGTTGAACTGTTCC-3′
VHH-Back	5′- GATGGGCTCTTCTGTGTGCAGCTGCAGGAGTCTGGRGGAGG-3′
VHH-Forward	5′-CTAGTGCTCTTCCGCTTGGAGACGGTGACCTGGGT-3′

Supplementary Table 5: The PCR protocol for the amplification of the primers.

**Cycle**	**Step**	**Temperature**	**time**
1 X	Initial Denaturation	95˚C	3min
35 X	Denaturation	95˚C	1 min
	Annealing	58˚C	1 min
	Synthesis	72˚C	1 min
1 X	Final Extension	72˚C	10 min
1 X		4˚C	-

Supplementary Table 6: The experimental conditions used to acquire data from the BLI.

Step	Name	Time (s)	Shake Speed
1	Sensor Check	30	1000
2	Loading (10 µg/mL CD16)	N/A	1000
3	Baseline (DPBS + 0.05% Tween 20)	120	1000
4	Association	300	1000
5	Dissociation (DPBS + 0.05% Tween 20)	600	1000

Supplementary Table 7: The mean fluorescent intensity (MFI) and percentages of the $CD16a^+$ and $CD16b^+$ cells that were labeled by anti-CD16a C1 VHH and 3G8 mAb are shown. The percentage and MFI of B cells that were labeled by C1 VHH and AT10 mAb are also shown. PE^+ and PE^- refers to the percent of the cells that were labeled with PE fluorescent dye.

**Name**	**NK92 Cells**			**Neutrophils**			**B Cells**		
	**PE+ (%)**	**PE⁻ (%)**	**MFI**	**PE+ (%)**	**PE⁻ (%)**	**MFI**	**FITC+ (%)**	**FITC⁻ (%)**	**MFI**
**Isotype**	0.71	99.3	25014	4.42	95.6	877	1.10	98.9	3.21
**3G8**	82.2	17.8	88225	61.09	38.91	18026	N/A	N/A	N/A
**C1**	79.9	20.1	91103	10.3	89.7	5175	1.74	98.3	4.21
**AT10**	N/A	N/A	N/A	N/A	N/A	N/A	94.6	3.61	78.2

Supplementary Table 8: The MFI and percentages of the HER2$^+$ and HER2$^-$ cancer cells that were labeled by anti-HER2 E5 VHH, trastuzumab, and pertuzumab are shown. PE$^+$ and PE$^-$ refers to the percent of the cells that were labeled with PE fluorescent dye. The total MFI of cells that were labeled with transtuzumab and pertuzumab appeared to be higher than VHHs. The reason is that the secondary antibody that was used to detect trastuzumab and pertuzumab was polyclonal against light and heavy chain (1 antibody/ multiple labels) resulting in a higher signal intensity. In contrast, the secondary antibody that was used to detect histag in VHHs was monoclonal (1 VHH/ 1 label) which generated lower signal intensity.

**Name**	**SKOV-3**			**MDA-MB-231**		
	**FITC+ (%)**	**FITC$^-$ (%)**	**MFI**	**FITC+ (%)**	**FITC$^-$ (%)**	**MFI**
**Isotype**	0.97	99	915	6.67	93.3	50
**Pertuzumab**	98.4	1.49	354125	68.9	31.1	3580
**Trastuzumab**	97.6	1.86	425871	67.5	32.5	5858
**E5**	93.3	6.45	106010	5.79	94.2	1989

Supplementary Table 9: The amino acid sequence of the BiKE:HER2/CD16a (E5C1 clones).

Name	Amino Acid Sequence
BiKE:HER2/CD16a<br>*(E5C1 clones)*	QVQLQESGGGLVQAGGSLRLDCAASGRTLSSYVVGWFRQAPG<br>KEREVVAAIGWSRTSTFYTDSVKGRFTISRDNTENTVYLQMNSL<br>KPGDTAVYYCAADSSPRRWDRESDFGSWGQGTQVTVSSPSGQ<br>AGAAASESLFVSNHASQVQLQESGGGLVQAGGSLRLSCAASGR<br>TFRLYRMGWFRQAPGKEREFVGSIKMIDGSTLYGDPVKGRFTIS<br>RDNTKFMAYLQMNSLKPEDTAVYYCASVSRVTGSYDSWGQGT<br>QVTVSSWELQGSEQKLISEEDLHHHHHHHHHHHH*

*E5 anti-HER2 VHH – HMA Linker – C1 anti-CD16a VHH – Protease Site – cMyc Tag – Histag

Supplementary Figures

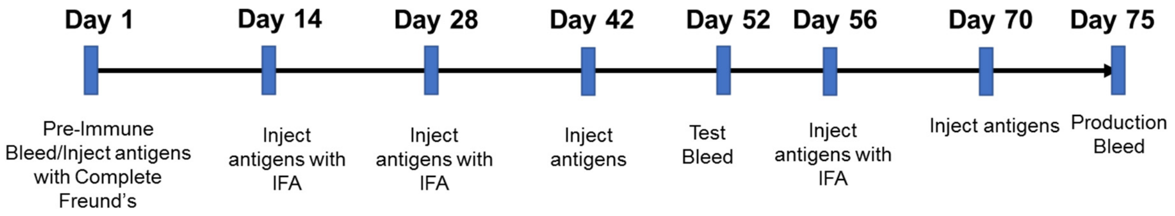

Supplementary Figure 1: The timeline and method used to immunize llama for the generation of VHHs against rCD16a and rHER2.

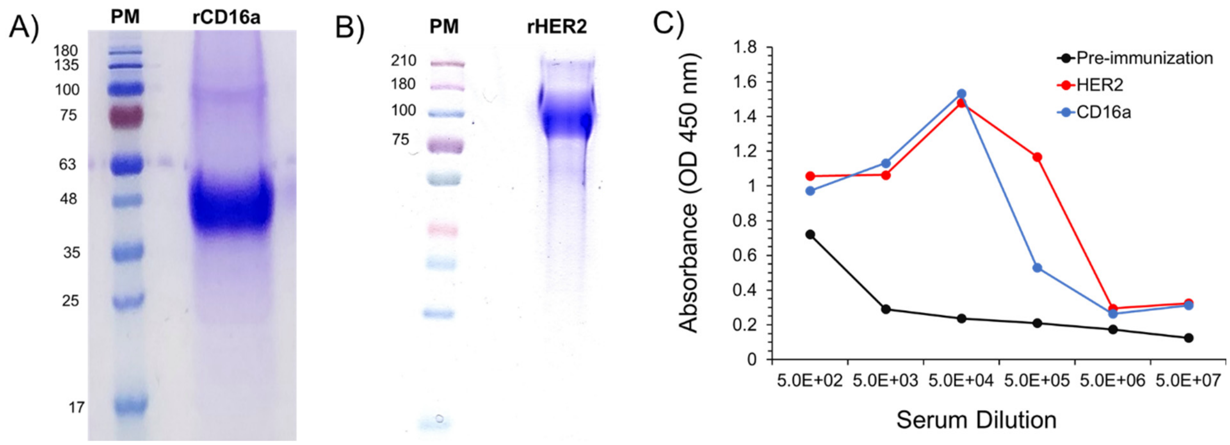

Supplementary Figure 2: A-B) The SDS-PAGE analysis of purified rCD16a and rHER2 (20 µg loaded). C) The level of IgG in serum of llama before and after immunization with rCD16a and rHER2 proteins as measured by ELISA.

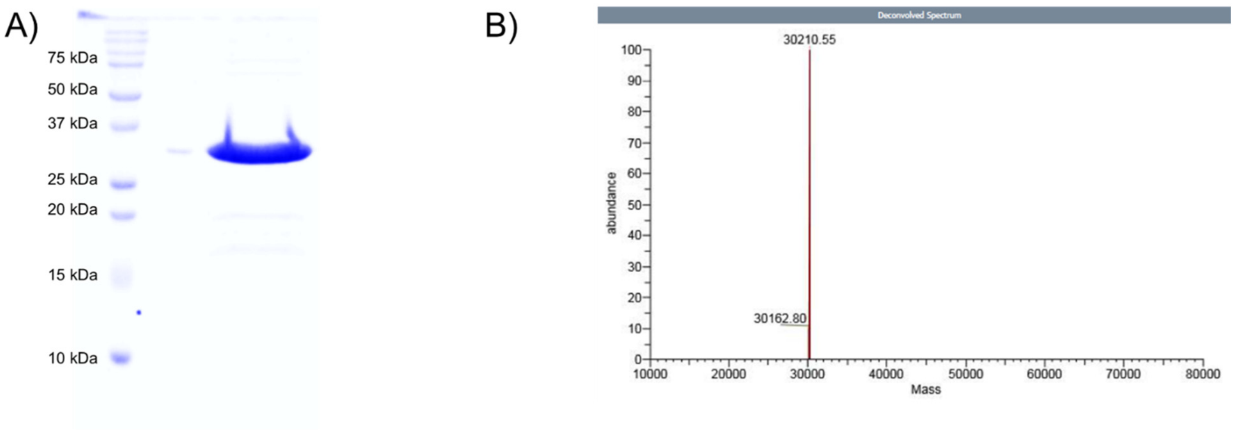

Supplementary Figure 3: A) SDS-PAGE analysis of the purified BiKE:HER2/CD16a with estimated purity of >95%. B) LC-MS graph of the purified BiKE:HER2/CD16a (~1 mg/ml) showing the peptide as monomer without the presence of dimer or multimer.

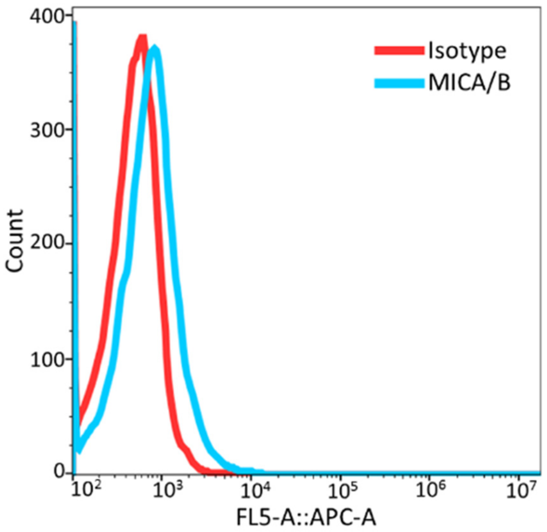

Sample	APC+	APC-	Mean	Count
Isotype	1.28	98.72	467	12854
MICA/B	7.53	92.47	717	12900

Supplementary Figure 4: Flow cytometry analysis of MICA/B expression on SKOV-3 cancer cells. This figure shows that the NKG2D ligands (MICA/B) have a very low expression levels on SKOV-3 cells.
